# The role of environmental constraints in walking: Effects of steering and sharp turns on gait dynamics

**DOI:** 10.1038/srep28374

**Published:** 2016-06-27

**Authors:** Dobromir G. Dotov, Benoît G. Bardy, Simone Dalla Bella

**Affiliations:** 1 EuroMov, Université de Montpellier, Montpellier, France; 2 Centro de Ciencias de la Complejidad (C3), Universidad Nacional Autónoma de México, Ciudad de México, México; 3 Institut universitaire de France (IUF), Paris, France; 4 International Laboratory for Brain, Music, and Sound Research (BRAMS), Montreal, Canada; 5 Dept. of Cognitive Psychology, WSFiZ, Warsaw, Poland

## Abstract

Stride durations in gait exhibit long-range correlation (LRC) which tends to disappear with certain movement disorders. The loss of LRC has been hypothesized to result from a reduction of functional degrees of freedom of the neuromuscular apparatus. A consequence of this theory is that environmental constraints such as the ones induced during constant steering may also reduce LRC. Furthermore, obstacles may perturb control of the gait cycle and also reduce LRC. To test these predictions, seven healthy participants walked freely overground in three conditions: unconstrained, constrained (constant steering), and perturbed (frequent 90° turns). Both steering and sharp turning reduced LRC with the latter having a stronger effect. Competing theories explain LRC in gait by positing fractal CPGs or a biomechanical process of kinetic energy reuse. Mediation analysis showed that the effect of the experimental manipulation in the current experiment depends partly on a reduction in walking speed. This supports the biomechanical theory. We also found that the local Hurst exponent did not reflect the frequent changes of heading direction. This suggests that the recovery from the sharp turn perturbation, a kind of relaxation time, takes longer than the four to seven meters between successive turns in the present study.

Healthy walking is characterized by a pattern of gait cycle durations that is variable but not random. When regarded as a time series, stride durations^1^ and other spatio-temporal parameters of gait such as stride length^2^ have a slowly-decaying auto-correlation function or long-range correlation (LRC). This means that a statistical dependence exists between each duration and the immediately subsequent duration as well as all later durations; a fluctuation in stride duration at a given moment has a lasting effect and affects all subsequent stride durations. LRC implies that the series exhibits the same statistical dependence on all temporal scales, analogous to geometrical self-similarity^3^. This phenomenon suggests that stride durations are controlled collaboratively in a distributed fashion by a large number of control degrees of freedom and that loss of observed LRC is likely to result from the loss of degrees of freedom due to a pathology^4^. This process is exemplified by stride durations that become increasingly random with aging^5^ and neurodegenerative disorders such as Parkinson’s disease (PD)^5^ or Huntington’s disease^6^. In this sense, loss of LRC can be interpreted as a marker of neuromuscular decline.

Loss of complexity in motor behavior has been associated generally with pathology, but can also occur due to externally imposed structure or when the number of functional degrees of freedom of the neuromuscular apparatus is reduced extrinsically^7^,^8^. For example, the complexity of isometric force production changes as a function of the required pattern of dynamic interactions with the environment^9^. Investigations comparing free tapping to metronome-cued tapping suggest that the effect of coordinating the timing of motor activity with an externally imposed structure is to reduce LRC^10^, probably because cued tapping is a sequence of corrections for deviations from the imposed structure.

Another possibility is that external fixed constraints impose a structure that substitutes for internal available degrees of freedom in a distributed system of control^11^. By “distributed system of control” we mean here that control is extended beyond the requirements of a specific task. For example, one might hypothesize that the timing mechanism for finger taps also covers other supporting aspects of the task such as maintaining arm posture and paying attention to the stimulus. This is not a trivial theoretical stance for one can also suppose that a localized mechanism dedicated uniquely to the timing of the finger is responsible for all effects.

Environmental constraints or perturbations may affect the measure of LRC because estimating LRC requires extended walking trials and in many cases the available conditions do not permit that. For example, setups requiring participants to make frequent 180 degrees turns at the end of a hallway have been used in the past^12^. Conceivably, walking in a narrow space such as a hospital corridor or turning around corners can also be considered as perturbations. The impact of external constraints on gait has been tested experimentally by way of treadmill walking^13^ and instructed metronome-paced (cued) walking^1^,^14^ where LRCs are typically found to decrease. In these situations, it is likely that external guides such as a treadmill or acoustic cues act as direct substitutes for parts of the intrinsic motor control of gait cycle timing.

Viewing LRC as the product of distributed control leads to additional predictions. Indirect constraints, namely those that do not act on the resulting output variable, should have a similar effect to a direct constraint as long as they use up degrees of freedom otherwise allocated for distributed control. This idea calls for more subtle manipulations. For example, increasing the difficulty of the task or using a secondary task executed in a different modality tends to be associated with a drop of LRC^15^. To this end, the first objective of the present study is to test whether an indirect constraint on gait cycle timing would result in a drop of LRC.

Gait regulation for steering is one such factor that does not directly determine step timing in the same way that instructed synchronization with a cueing stimulus would^13^. In this way it complements previous studies such as ones involving treadmill walking where the constant constraint of keeping fixed velocity and a limited stepping range results in reduced LRC specifically in stride velocities. It is expected that constant steering as a form of constant constraint on performance will lead to a decrease in LRC. During steering, gait produces destabilizing sideways forces. This is so because, first, a relatively constant centrifugal force vector points away from the direction in which the feet are falling; second, moment profiles of lower extremity joints increase in proportion to steering angle^16^; and third, center of mass is located above these joints. Not surprisingly, steering is a skill involving an anticipatory strategy that young children acquire only after first having acquired straight walk abilities^17^. Furthermore, steering is oftentimes the first mode of walking to be degraded in the course of progression of certain motor disabilities caused by neurological dysfunction (e.g. cerebral palsy^18^) and neurodegenerative disorders (e.g. PD^19^). In the latter case, turning can even be a trigger for freezing of gait (FOG)^19^.

Arguably, LRC endows physiological control processes with an increased resistance to external perturbations^4^,^20^. Yet, such processes cannot be completely immune to external interference. In fact, perturbations consisting of altering the functional linkage between movement and task performance can reduce or destroy altogether the LRC properties of the given motor behavior^21^,^22^. Consequently, external perturbations of gait are likely to reduce LRC. Executing a sharp turn without interrupting gait is a manoeuvre even more challenging than continuous steering. Hypothetically, occasional 90 degrees turns can be categorized as perturbations of gait dynamics. Several muscles become engaged in order to reduce forward speed^16^ and contribute to medio-lateral center of mass acceleration during sharp turns^23^.

To summarize, the main goal of the present study is to investigate, first, the effect of steering as a stationary constraint on performance and, second, the effect of sharp turns as perturbations of performance. The ultimate purpose of determining that task constraints alter LRC is twofold with both methodological and theoretical implications. On the methodological side, studies performed in varying conditions involving different environmental constraints have to be compared with caution. For example, if in a clinical study patients are asked to walk in a narrow corridor or make frequent turns this might lead to reduced LRC relative to other “benchmark” studies where healthy participants walked on a large athletic track. Yet, this difference may not be a consequence of the disease, but it might rather be due to the different environmental constraints. On the theoretical side, further support is needed for the hypothesis that LRC in gait and motor performance in general is reduced by indirect constraints that limit the internal degrees of freedom.

The present study used the LRC of inter-stride-intervals as principal dependent variable. This is motivated by the fact that this is a relevant measure, it is frequently reported in the literature, and is also the most reliable and accurate to obtain in ambulatory conditions. Unperturbed and unconstrained walking on a large athletic track was the control condition against which two other conditions were compared. In the constantly constrained condition, an oval (roughly elliptical) trajectory imposed a constant low-angle steering mode of walking and was expected to decrease LRC (indirect constraints, Hypothesis 1). In the perturbed condition, a rectangular trajectory implemented straight-line walking interrupted by frequent 90 degrees turns and was expected to also lead to a decrease of LRC (perturbation, Hypothesis 2). It is difficult to predict based on the general theoretical considerations which of the two manipulations would have a stronger effect. These circumstances of performance also address the general question: How is gait affected by real-world environmental constraints that are typical in realistic situations but rarely investigated in the laboratory?

Next, two more questions with theoretical significance are worth investigating within the span of the present design. The temporal evolution of the immediate effect of sharp turns could shed light on the way gait dynamics recover from perturbations. To determine such potential relationship, the degree of turning in each stride was correlated with a local measure of LRC calculated per stride based on methods proposed for determining the instantaneous analogue of LRC^24^,^25^. A correlation between degree of turning and local LRC is expected if the typical time to recover from a perturbation from a sharp turn is less than the average period until the subsequent perturbation (recovery time, Hypothesis 3).

Processes in stride durations that appear to have long-memory (LRC) could possibly have a biomechanical origin^26^,^27^. Maintaining a smooth exchange of kinetic energy and potential energy is essential for efficient walking^28^. This allows a certain proportion of the kinetic energy to be re-used, up to about 65%^29^. Such efficiency is essential for upright locomotion and, consequently, the discovery of this pendulum dynamic is a significant landmark in early locomotory development^30^. Ahn and Hogan^26^ have made the connection between this energy re-use and a type of self-sustaining dynamics that slowly forgets perturbations resulting from neuromuscular noise, thus reproducing the slowly decaying autocorrelation typically referred to as LRC. Importantly, kinetic energy depends proportionally on velocity and for this reason changes in average velocity across trials imply changes in the average amount of kinetic energy re-used among gait cycles. Consistent with this linkage, LRC has been found to increase in treadmill walking as velocity increased beyond the self-selected^7^,^31^ (although it also increased in the other direction). In those studies, the *α* exponent from detrended fluctuation analysis^32^ was used as a measure of LRC, whereby *α* < 0.5 indicates anti-persistence, *α* = 0.5 indicates randomness, and 0.5 < α ≤ 1 indicates LRC. The same measure was employed in the present study because it is the most widely used method to estimate LRC from empirical data and in this way it would facilitate the comparison with other studies.

To further test this model within the experimental protocol adopted in the present study, we investigated the relation between LRC and overground walking velocity using *mediational analysis* (or covariance structure analysis), a technique targeted at hypothetical causal relations among observed variables. Accordingly, a part of the variation in LRC in gait due to the experimental manipulation is expected to be mediated by changes in velocity where increase in self-selected velocity leads to increase in LRC (biomechanical basis of LRC, Hypothesis 4).

## Results

### Turning magnitude

**Figure 1:**
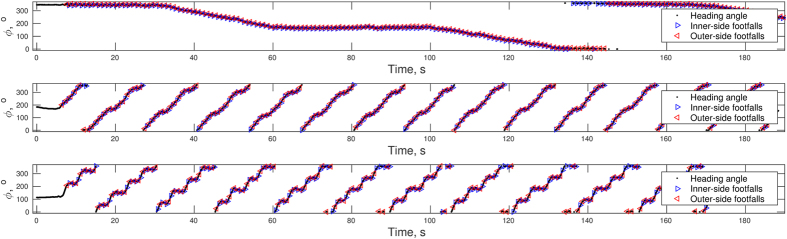
Sample continuous heading angle recordings along with footfall timing are shown for unconstrained walking on the track (top), constrained constant steering on an elliptical path (middle), and sharp turning on a rectangular path (bottom).

**Figure 2:**
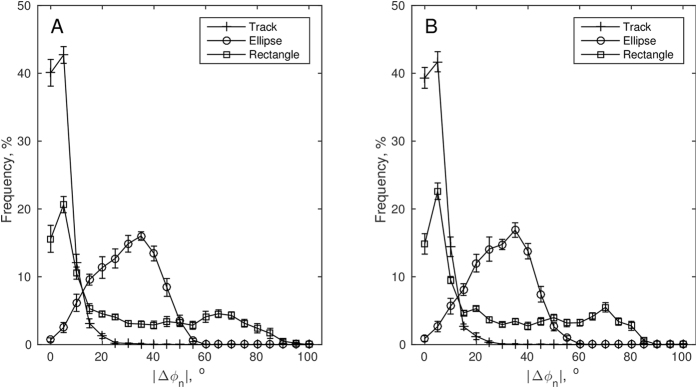
Distributions of inner-side (**A**) and outer-side (**B**) absolute inter-stride-heading-angle-differences. Shown are bin averages (*SE*) for the histograms computed in each trial. The three experimental conditions are identified here in terms of the respective shapes of the pathway: track (unconstrained), ellipse (constrained), and rectangle (perturbed).

After reducing the continuous heading angle, Fig. 1, to absolute inter-stride-heading-differences |Δ*φ*_*n*_|, or simply turning magnitude, the averaged histograms in Fig. 2 suggest that both for inner-side and outer-side strides the distributions of the turning magnitude were unimodal in the track and elliptical conditions but bimodal in the rectangle condition. For inner side turning magnitudes, the unimodality null hypothesis could not be rejected by Hartigans’ dip test, *D* = 0.0045, *p* = 1, and *D* = 0.0059, *p* = 0.96 for track and constant steering, respectively. For sharp turning, the unimodality null-hypothesis was rejected, *D* = 0.0141, *p* < 0.05. Similarly, for outer side turning magnitudes, the unimodality null-hypothesis could not be rejected, *D* = 0.0058, *p* = 0.94, and *D* = 0.0052, *p* = 1 for track and constant steering, respectively, while for sharp turning the null hypothesis of unimodality was rejected, *D* = 0.0157, *p* < 0.01. As Fig. 2 shows, turning magnitude was distributed mostly around 5 degrees for walking on the track. On the constant steering path, the mode was around 35 degrees and the distribution was wider. On the sharp turning path, the first mode was found around 5 degrees consistent with straight walking and the second one around 70 degrees consistent with sharp turning.

### Average gait cycle parameters and LRC

Preliminary statistical analyses did not indicate any effects of or interactions with turning direction. For this reason, in order to simplify the results, trial outcomes were averaged across left and right turns and the analyses reported in this section are based on this averaged data. The spatio-temporal gait parameters grouped per experimental condition are summarized in Table 1. The table also summarizes the results of the ANOVAs and condition comparisons. As can be seen, LRC estimated using the *α* exponent decreased significantly from the Unconstrained to the Constrained condition, and decreased further from the Constrained to the Perturbed condition. Velocity was affected similarly by the conditions. In addition, both ISI and CV increased in Constrained relative to Unconstrained, and increased further in Perturbed relative to Constrained. The same pattern of effects was observed in the inner-side and outer-side measurements.

### Association between steering and turning and spatio-temporal parameters of gait

**Figure 3:**
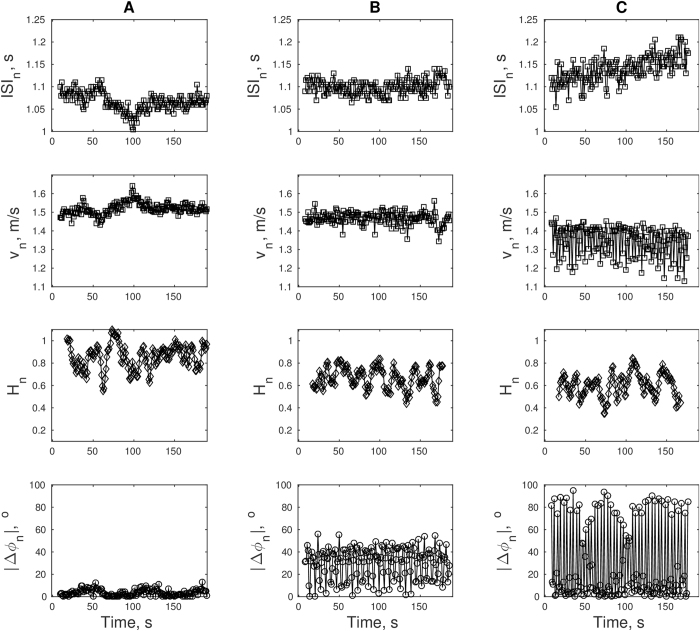
Series of inter-stride-intervals (top), velocities (second row), local Hurst exponents (third row), and absolute inter-stride-heading-angle-differences (bottom) are shown from three sample trials in the (**A**) unconstrained, (**B**) constant steering, and (**C**) perturbed conditions.

Correlations between the turning magnitudes series |Δ*φ*_*n*_| and the time series for selected gait parameters were obtained in each trial (see Fig. 3 for sample time series). In particular, correlations between |Δ*φ*_*n*_| and the parameters ISI, *v*, and SL were obtained (summarized in Table 2). As can be seen in Table 2, the correlation between |Δ*φ*_*n*_| and ISI tended to increase from Unconstrained and Constrained to Perturbed. This effect was statistically significant for the outer-side measurements but not for the inner-side measurements. Similar trends were observed for the magnitude of the correlations between |Δ*φ*_*n*_| and *v* and between |Δ*φ*_*n*_| and SL but these trends were not associated with statistically significant effects of condition.

### Immediate effect of steering and turning on the local Hurst exponent

The correlation coefficient between the turning magnitude |Δ*φ*_*n*_| series and local *H*_*n*_ series was obtained for each trial (see Fig. 3 for sample series). For inner side measurements, about 57% of the trials in unconstrained walking resulted in significant correlations albeit very small in magnitude: the average coefficient was <*r*> = −0.087. For outer side measurements, about 36% of the trials in unconstrained walking resulted in significant correlations with a modest average coefficient of <*r*> = −0.082. No trials in constrained and perturbed conditions produced significant correlations between |Δ*φ*_*n*_| and *H*_*n*_.

### Covariance structure analysis for determining the influence of gait velocity on LRC

The correlation matrix presented in Table 3 shows strong associations among the selected parameters and high consistency among the inner and outer side measurements. In particular, the association between *α* and velocity, together with the observation that condition affected both *α* and velocity (Table 1) motivates a mediation analysis. In this analysis, the effect of condition on *α* is separated into two pathways: direct and indirect. The direct pathway is conceptually similar to the main effect of the ANOVA. The indirect pathway reveals how much of the effect of condition on *α* is due to the effect of condition on velocity.

To facilitate interpretation, velocity was centered to an unconstrained control trial separately for each participant (v*_ij_* = v*_*ij*_*–v*_*Unconstrained,j*_). In order to avoid a possible transient effect from a first trial in the session, the participant’s second control trial was used. In this way, the intercept of the model for *α* (indicated by *γ*_*α*_) can be compared to the means shown in Table 1. The results provided by a simple model (without the mediating factor), and by a second mediated model, are reported in Table 4. The outcomes of the mediation modeling were similar for inner- and outer-side measurements.

First, the simple model for *α* is *α*_*ij*_ = (*γ*_*α*_ + *ζ*_*α,j*_) + (*c* + *ζ*_*c,j*_) Condition_*ij*_ + *ε*_*ij*_ . This model confirms the results of the repeated-measures ANOVA, see Table 1. Note that when comparing the estimates in Table 4 with the means reported in Table 1, *γ*_*α*_ corresponds to the mean *α* in the Unconstrained condition while *c* corresponds to the amount of drop in *α* as a function of condition. *ζ* are random effects.

Second, the mediated multilevel model, including velocity as a mediating variable is:


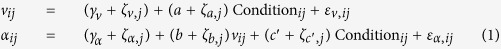


**Figure 4:**
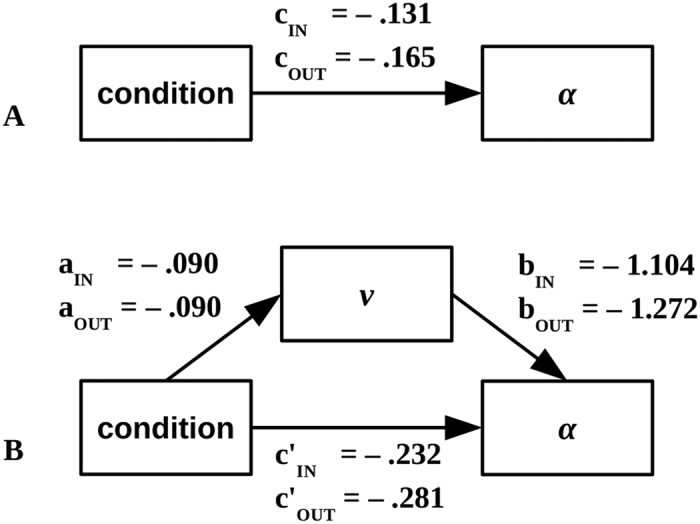
Schematic illustration of the unmediated effects (**A**) and mediated effects (**B**) of path trajectory (Condition) on *α* via velocity.

As can be seen in Table 4, the second model reveals a slightly more complicated pattern. The estimated coefficients *a* and *b* indicating the *indirect* effect of *α* both reached significance. The results of the mediation analysis are illustrated in Fig. 4 where the simple and the mediated pathways are shown in parallel. As expected in a pathway analysis model, *c*′ + *ab*≈*c*, the sum of the effects of the direct and indirect pathways is approximately equal to the effect found in the simple, non-mediated model. Note that the *direct* effect of condition (*c*′) in the mediated model is clearly more negative than in the non-mediated model (*c*′ < *c*). In contrast, the indirect effect of condition (*ab*) is positive due to the positive dependence of LRC on velocity, a bit of information that cannot be obtained directly neither from the ANOVAs nor from the correlation tables. (Both b and γ_V_ are negative in Table 4 and their product makes for a positive effect in Eq. 1).

## Discussion

In the present study we investigated the effect of a constant constraint and occasional perturbations on gait dynamics. Additionally, we examined the dependence of LRC on gait velocity. As expected (Hypothesis 1), LRC in stride-to-stride control was reduced by environmental constraints that engaged additional neuromuscular degrees of freedom, i.e. steering associated with increased moment in the lower extremity joints^16^ and anticipatory head tilt^17^. In this sense, we find support for the theory that fixing or recruiting the available degrees of freedom that are non-obviously related to the actually measured performance variable can nonetheless reduce LRC in the overall behavior^11^. Consequently, alterations in LRC can result either from pathology or task constraints^9^.

Interestingly, these factors can be similar in nature and hard to disentangle in the cases where the mechanism of a given pathology is to reduce the available degrees of freedom. Unfortunately, it is also difficult to distinguish between the effects of pathological state and constraints when they are mixed because of a potential flooring effect. The theoretical result of strongly constraining internal resources or perturbing performance is a random time series, not anti-persistent intervals as is the case in externally cued tapping. For this reason, if a manipulation is strong enough, i.e. the demands on precision are beyond the normally possible, all participants can become equally random in their motor performance regardless of their health status.

We found that perturbations of walking also deteriorated LRC (Hypothesis 2), more so than the environmental constraint. Notice that one could have predicted the opposite effect in the current study for methodological reasons. Superimposed periodic trends are known to lead to LRC overestimation^33^. By the design of the pathways, participants were expected to change dramatically their gait at the moment of turning. In particular, it was expected that there would be little steering when participants were walking on the track, relatively constant steering in the elliptical condition, and a succession of straight walking and sharp turning in the rectangle condition. Indeed, the distribution of heading angle differences per stride supports the existence of a bimodal kind of steering behavior only in the sharp turn (Perturbation) condition.

There was modest evidence for an association between turning magnitude and spatiotemporal parameters of gait such as ISI, velocity, and stride length. The correlations were very low and only in one case (ISIs taken from outer-side) did they appear to depend on experimental condition whereby ISI tended to increase with increases of turning magnitude in the Perturbation condition. The immediate effect of environmental influences on gait dynamics was also not apparent in the correlations between turning magnitude and local Hurst exponent in the Constrained and Perturbed conditions; Hypothesis 3 was not supported. Taking into account the main effect on the global *α* exponent, the conclusion is that the time for recovery from the sharp turn perturbations, a sort of relaxation time, is longer than time to reach the subsequent perturbations. In the current design, this corresponded to the time to cover the distance between the corners of the rectangle, i.e. between seven and four meters.

Interestingly, sharp turns are particularly problematic for patients with advanced PD and oftentimes act as a trigger of FOG episodes^19^,^34^. A neuroimaging study reveals that this may be the result of reduced sensorimotor integration^35^. More generally, motor switching (e.g., changing walking direction in response to an external cue), has been associated to FOG^36^. To date, the precise mechanism underpinning this effect is not known. The present results may suggest an additional perspective on this intriguing phenomenon and pave the ground to further investigations of the nature and onset of FOG using dynamical systems theory. According to this approach, walking and standing still can be thought of as two principle dynamical modes of controlling upright stance. External perturbations may provoke spontaneous transitions among these modes. If one mode (e.g., standing) is more stable relative to the other (e.g. walking), then a perturbation is likely to result in a spontaneous transition to the more stable mode.

This is consistent with growing neurophysiological evidence indicating that PD is a dynamical disease consisting of stable mode-locking patterns in the basal ganglia^37^. In particular, strongly coherent periodic firing emerges in the globus pallidus (GPe)-subthalamic nucleus (STN) network, with the end result being to block the flexible transmission of information in the sensorimotor pathways passing through the thalamus^38^. That basal-ganglia thalamo-cortical circuitry may underpin the control of correlated timing fluctuation is suggested by a recent observation in a single case showing that deep brain stimulation targeted at the subthalamic nucleus does improve LRC^39^.

Providing evidence that sharp turns act as perturbations in healthy adults contributes to this dynamical theory of gait mode switching. Accordingly, sudden strong changes of environmental constraints on walking act as perturbations on neural motor control. Under this interpretation, the parkinsonian brain is unable to adaptively accommodate such perturbations and in consequence they are likely to push the patient off of the dynamic mode corresponding to walking and into a standing still episode which corresponds to the most stable, mode-locked state. In this respect, a separate clinical investigation by our team explores how an adaptive cueing technique might help PD patients maintain the properties of healthy gait.

The design of the present experiment also allows to discuss the provenance of LRC in gait dynamics. The most promising theories explaining this phenomenon can be categorized into two groups. In the one group, several different models repose on the so-called central pattern generators endowed with fractal properties^40^,^41^,^42^. According to these ‘fractal CPG’ models, the reason why gait exhibits LRC is because the central pattern generators in the central nervous system possess LRC properties. Contrary to the case of invertebrates, however, the evidence for the very existence of CPG in human locomotion is either indirect or even contradictory^43^,^44^. Another group of theories accounting for LRC in stride-to-stride variability links the slowly decaying autocorrelation to the biomechanics of walking^26^,^45^ and, in particular, the inter-stride re-use of kinetic energy.

A portion of the reduction in LRC across all conditions was mediated by a reduction in walking velocity (Hypothesis 4). Because mediation analysis imparts a causal role to the mediating variable, the effect counts as empirical support for the biomechanical basis of LRC. In this sense, the biomechanical models^26^,^45^ gain advantage over complex CPG models^42^ because the latter do not hold an obvious explanation why the statistical properties of the CPG should depend on body dynamics, although they can accommodate the pattern of results by way of setting parameters post-hoc.

**Figure 5:**
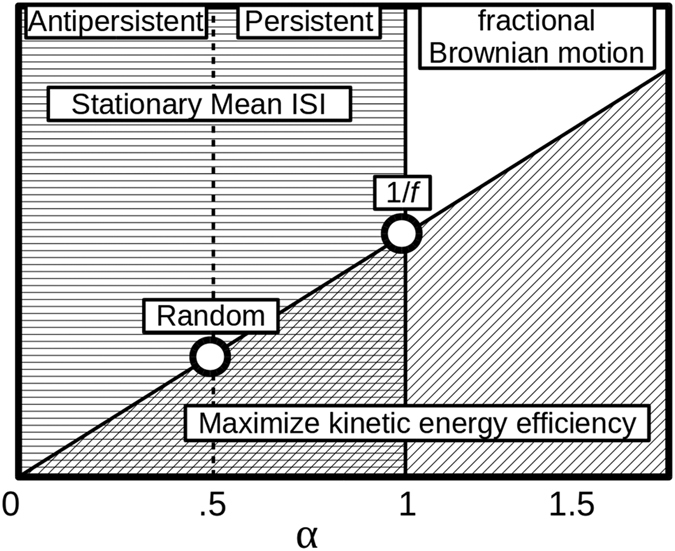
Map of the LRC parameter *α* and constraints on stride-to-stride variability.

A final clarification of the proposed biomechanical model of walking dynamics^26^ is needed here. Note that Ahn and Hogan’s model does not explain why ideal LRC (1/*f* noise) is the *optimal* kind of variability for gait cycle parameters, only that memory is an increasing function of energy efficiency. The model predicts that *α* > 1 (strong local memory characteristic of Brownian motion) should correspond to even better performance as compared to 1/*f* noise. We can amend this by taking into account another important relation that derives from the pendulum dynamics of human walking^28^,^46^,^47^,^48^. Gait cycle possesses a characteristic frequency and, consequently, stationary mean period. Stationarity is a property of fluctuations limited to the fractional Gaussian noises, thus not exceeding in the range of fractional Brownian motions, hence *α* ≤ 1^49^,^50^. As expressed schematically in Fig. 5, superimposing this theoretical relation and the biomechanical model results in a maximum at *α* = 1 or 1/*f* noise.

Clinical evidence concerning central and peripheral neural pathology also favors the biomechanical approach versus the fractal neural CPG theories. LRCs are preserved in the earlier stages of Parkinson’s disease albeit degeneration in the basal ganglia and movement impairments are expressed clearly^51^. A substantial neural loss in the basal ganglia has typically already occurred in the early stages of Parkinson’s disease when patients receive the first diagnosis^52^ and cardinal motor symptoms such as reduced stride length and reduced gait velocity are already observable at that stage^19^. LRC is still intact, however, despite neural degeneration. Similarly, peripheral nerve degeneration does not seem to affect LRC in gait but it affects basic spatio-temporal parameters^27^. In sum, LRC seems to be a stable property of gait as long as the walker is capable of maintaining a steady gait.

A measurement problem related to the constancy of boundary conditions in walking trials needs to be discussed following the findings presented here. Because the empirical *α* exponent in gait trials is sensitive to environment-driven constraints, particular attention should be paid when comparing results from different studies given that the experimental settings might differ widely. Ideal unconstrained conditions of performance are difficult to achieve in a laboratory setting. The reliable estimation of scaling exponents demands a large number of strides performed under stationary boundary conditions^53^.

In practice, only a large athletic track would allow one to walk for an extended period of time without being perturbed or without encountering a substantial environmental variation. This appears to be the exception rather than the norm in everyday life. In comparison, walking indoors very often involves making frequent sharp turns. In fact, the idealized conditions necessary for an un-biased test of LRC in gait are difficult to obtain. For the purpose of the argument, consider idealized testing conditions. A participant moving at a speed of 1.5 m/s would be approaching a kilometer of distance traveled after only ten minutes of walking. Even if a large open area with constant parameters (slant, surface material, etc.) existed and was available for testing, it is likely to involve a variety of environmental constraints such as passing cars or people, changing illumination and changing optic flow if the space were inside a large mall. In sum, it seems difficult to avoid the effects of environmental constraints on gait dynamics apart from very controlled and thus artificial experimental conditions (e.g., walking on a treadmill). At the same time, those constraints cannot be ignored if the results obtained in the laboratory have to be generalized to everyday walking, especially indoors walking.

Beyond these methodological limitations, our findings suggest that LRC in ISI (but very likely in other parameters of gait and other motor skills as well) is never governed by the motor system only, i.e. by an encapsulated mechanism and deprived of external constraints. To the contrary, LRC in gait is the expression of the dynamical interactions between the motor system and the environment. Hence, a particularly sensitive test of the functioning of the motor system is not about the performance in optimal circumstances but rather the flexibility with which an individual adapts to environmental constraints. Estimation of LRC in a trial involving free walking on the track would constitute an example of the former while the degree to which LRC is affected by varying task constraints and perturbations would constitute an example of the latter.

## Methods

### Participants

Seven healthy young adults aged between 21 and 41 years (*M* = 29) without gait disorders volunteered to participate in this study. The study was part of a larger protocol approved by the local institutional review board (Comité pour la Protection des Personnes, Nîmes, France) and carried out in accordance with the approved guidelines. Each subject provided informed consent prior to participation.

### Materials and Procedure

The experiment was conducted in a reserved indoor track and field arena where each participant was submitted to three walking conditions. In the *Unconstrained walking* condition (control condition), participants walked on the athletic track (about 200 m). In the *Constrained, continuous steering* condition participants walked around an ellipse (6 × 3.6 m) delineated with cones on the ground. In the *Perturbed walking* condition participants walked inside a rectangular area (7 × 4 m) defined using cones on the ground. One round on the elliptical and rectangular paths approximated 20 m.

Small inertial measurement units (IMUs, 6DOF, 128 Hz) strapped over the left and right phalanges of the feet, anterior side of left and right tibia, and sternum collected acceleration and angular velocity during walking. After installation of the sensors and brief familiarization with the setup, participants performed two trials (left and right turning) in each condition. A total of six trials were performed, each with a duration of three minutes. Turning direction and order of conditions were randomized across participants. The experiment including preparation lasted approximately 30 minutes.

### Parameters of gait

Proprietary algorithms of the data collection system (MobilityLab, APDM Inc., Portland, OR) estimated spatio-temporal gait parameters and provided series of left and right stride durations, called inter-stride-intervals (ISI, s), stride lengths (SL, m), stride velocities (*v*, m/s) and their respective trial averages. Cadence and coefficient of variation (CV), which equals the SD of the ISI divided by the mean ISI, were computed. Left and right step timing corresponds to footfalls and was determined using standard algorithms that use the impulses recorded by IMUs on the feet and tibia. From these, ISI, CV, and cadence were calculated. For stride lengths and velocities, the data collection system employs a novel method to integrate the IMU data. Zero-velocity update allows to subtract integration error on a stride-by-stride basis and in this way to obtain reliable stride length and velocity data^54^,^55^,^56^. Separate testing (not reported here) inside a motion capture lab revealed that the trial averages of the estimated stride lengths and velocities approximate very well the optical motion capture “gold standard”.

Ideal LRC in the time domain corresponds to 1/*f* or pink noise in the power spectrum. The *α* scaling exponent (*α* = 1 for 1/*f* noise) estimated using *detrended fluctuation analysis* (DFA)^32^ has become the preferred measure of LRC because it performs better when certain kinds of slow non-stationarities are present in the signal. In empirical studies, LRC in gait^1^,^14^ oftentimes does not reach the theoretically ideal values but stays in the range of the so-called 1/*f*-like noise between white and pink noise (0.5 < *α* < 1).

LRC was estimated in terms of the *α* scaling exponent of each trial. To this end, DFA^32^ was applied to the series of stride durations (ISI) separately for inner-side and outer-side strides. Standard parameters were employed in the analysis: first-order detrending and a window size range from four points to a quarter of the time series. (Supplementary Figs 1 and 2 contain plots of the DFA fluctuation functions and corresponding linear fits for each trial.)

### Statistical Analyses

Variables with laterality (i.e., stride length) could potentially be affected differentially by turning. To control for this, analyses were performed separately for the interior or *inner-side* (IN) and exterior or *outer-side* (OUT) strides, where inner and outer were defined relative to the direction of the turn in the given trial. Repeated-measures ANOVAs tested for effects of experimental condition on spatio-temporal gait parameters including LRC. When necessary, conditions were compared with Bonferroni-corrected pairwise post-hoc tests. Associations among variables were assessed with Pearson’s correlations calculated across all participants and trials. All correlations and ANOVAs were repeated twice in order to analyze IN and OUT measurements separately.

### Mediation analysis

A statistical technique based on the structure of covariances among observed variables—*Mediation analysis*—was used to investigate the pattern of inter-dependence among parameters of gait and the role of average stride velocity in particular. This technique goes beyond correlations and allows evidence for hypothesized causal influences to be extracted from the covariance structure of a set of variables. Linear mixed-effects modeling extended for mediation analysis in a multi-level design was applied following recent recommendations for repeated-measures designs^57^,^58^ and using a statistical package^59^ for R. Two separate analyses (for IN and OUT) were performed with *α* as outcome variable. Level-1 predictors consisted of Condition and velocity as a potential mediator of the effect of Condition. Participants acted as Level-2 grouping variable (i.e. repeated measures). Condition was coded as a time-varying predictor equal to zero in unconstrianed trials, 1 in constrained trials, and 2 in perturbed walking trials. For the purpose of obtaining easily interpretable coefficients, the unit difference in the predictor levels means that the coefficient estimated for Condition should approximate the average change from Unconstrained to Constrained and from Constrained to Perturbed obtained in the usual way of analysis of variance.

According to Hypothesis 4, LRC depends on the biomechanics of kinetic energy reuse. For this reason, and also to avoid overfitting, the mediation analysis focused on the potential role of velocity and not the other parameters of gait. Preliminary analysis revealed that stride length just as *v* exhibits a correlation with *α* (see Table 3). Yet, velocity is a primary candidate for a mediating variable because it tests a theoretically motivated hypothesis.

### Inter-stride heading angle difference to measure turning magnitude

The trunk heading angle was used to verify that turning and steering behavior matched the intended design of the experiment. The orientation of the IMUs in a coordinate system aligned with the initial setup of the IMUs was calculated by integrating the gyroscopic angular velocity using the proprietary algorithm of the data collection system. In this way, a heading angle *φ* can be obtained in terms of the trunk IMU yaw—orientation in the horizontal plane. Sample continuous heading angle recordings from one trial in each condition are shown in Fig. 1 along with superimposed footfall times.

A suitable measure of the amount of steering consists of the absolute differences between successive heading angles taken separately at the time of footfalls, |Δ*φ*_*n*_|, where *n* stands for the the time of footfall *n*. The absolute inter-stride-heading-angle-differences quantify how much the walker has changed her overall orientation between two successive stable anchor points (placing the same foot on the ground). This reduction of the continuous heading angle allows the absolute inter-stride-heading-angle-differences to be compared directly with the ISI used in LRC calculation and with the local Hurst exponent.

To further verify the effectiveness of the manipulation, the turning magnitude distributions were obtained. It was expected that absolute steering angles would make a bimodal distribution in the sharp turning condition where participants alternate between two different modes of walking—straight line walking and sharp turning. Hartigans’ dip test addresses the null hypothesis that a given distribution contains a single mode. The dip statistic *D*_*k*_ quantifies the maximal distance between the empirical cumulative distribution and fitted upper and lower bound unimodal cumulative distributions. A significance test is obtained using a bootstrapping procedure. Here, Hartigans’ dip test with *k* = 10^3^ bootstrap iterations was applied to the |Δ*φ*_*n*_| pooled across all trials and participants. The test was performed separately for each condition and side.

### Determining how steering and turning affect ISI scaling by way of the local Hurst exponent

Typically, the scaling properties of noisy behavioral data such as ISI are described in terms of a Hurst exponent. This characterization implies that the data is an everywhere-singular time series. In theory, the scaling structure of such a series can be described locally as well. Each point is regarded as a singularity—the center of a fractional power of time—and the series of singularities can be quantified in terms of a series of singularity coefficients covering the whole data^25^. This characterization is desirable because it may contain information about changes of performance through the trial. A wavelet-decomposition method has been made available for this purpose^25^. More recently, a modified version of DFA has been proposed^24^ and it promises to capture the local scaling structure of a fractal time series in terms of local Hurst exponents. In the context of the current study, the series of local Hurst exponents can be used to determine if local scaling reflects the immediate effect of perturbations in the sharp turning trials and potential non-constant steering in the constrained trials. To this end, we applied the modified DFA^24^ using the author’s freely available toolbox and correlated the series of local Hurst exponents and turning magnitude |Δ*φ*_*n*_| in each trial. Ihlen’s method is also designed to address an additional issue, namely the multi-fractal properties of the signal. This is outside of the scope of the current study. For this reason, we obtained a single local Hurst exponent *H*_*n*_ per time point by averaging across the scales of the analysis. In order to achieve useful resolution and localization, relatively short windows from 2^2^ to 2^4^ were used. Exhaustive analyses showed that the results reported here do not depend on which scale of *H*_*n*_ calculation the correlations with |Δ*φ*_*n*_| was based on.

## Additional Information

**How to cite this article**: Dotov, D. G. *et al*. The role of environmental constraints in walking: Effects of steering and sharp turns on gait dynamics. *Sci. Rep*. **6**, 28374; doi: 10.1038/srep28374 (2016).

## Supplementary Material

Supplementary Information

## Figures and Tables

**Table 1:** Summary statistics, main effects, and Bonferroni-corrected pairwise comparisons for the gait parameters averaged across left and right turns.

		Unconstrained		Constrained		Perturbed		Main Effect		Comparisons		
		*M*	(*SD*)	*M*	(*SD*)	*M*	(*SD*)	*F*(2, 12)	*p*	U:C	U:P	C:P
IN	*α*	0.85	(0.150)	0.71	(0.135)	0.59	(0.117)	13.32	<0.001	<0.05	<0.001	<0.01
	ISI (s)	1.08	(0.042)	1.14	(0.057)	1.17	(0.052)	52.38	<0.001	<0.001	<0.001	<0.001
	CV (%)	1.55	(0.255)	1.80	(0.441)	2.66	(0.647)	31.89	<0.001	=0.07	<0.001	<0.001
	*v* (m/s)	1.46	(0.064)	1.35	(0.090)	1.28	(0.081)	126.40	<0.001	<0.001	<0.001	<0.001
	SL (m)	1.58	(0.086)	1.54	(0.086)	1.50	(0.088)	37.19	<0.001	<0.001	<0.001	<0.001
OUT	*α*	0.86	(0.126)	0.68	(0.124)	0.53	(0.105)	21.39	<0.001	<0.05	<0.001	<0.01
	ISI (s)	1.08	(0.042)	1.14	(0.057)	1.17	(0.052)	52.25	<0.001	<0.001	<0.001	<0.001
	CV (%)	1.53	(0.221)	1.84	(0.444)	2.90	(0.619)	38.21	<0.001	<0.05	<0.001	<0.001
	*v* (m/s)	1.46	(0.064)	1.35	(0.091)	1.29	(0.081)	126.38	<0.001	<0.001	<0.001	<0.001
	SL (m)	1.58	(0.086)	1.54	(0.087)	1.50	(0.089)	37.15	<0.001	<0.001	<0.001	<0.001

All gait parameters were estimated separately on the inner- (IN) and outer-side (OUT) of the walker with respect to the direction of the turn.

**Table 2:** Summary statistics, main effects, and Bonferroni-corrected pairwise comparisons of the correlations between turning magnitude and selected gait parameters in the three experimental conditions.

		Unconstrained	Constrained	Perturbed	Main Effect		Comparisons		
		<*r*>	<*r*>	<*r*>	*F*(2, 12)	*p*	U:C	U:P	C:P
IN	|Δ*φ*_*n*_| ~ ISI	0.19	0.18	0.32	1.37	0.290	–	–	–
	|Δ*φ*_*n*_| ~ *v*	−0.08	−0.14	−0.14	<1	–	–	–	–
	|Δ*φ*_*n*_| ~ SL	0.01	−0.11	−0.12	3.37	0.070	–	–	–
OUT	|Δ*φ*_*n*_| ~ ISI	0.20	0.27	0.44	3.39	0.056	0.60	0.054	<0.01
	|Δ*φ*_*n*_| ~ *v*	−0.08	−0.11	−0.11	<1	–	–	–	–
	|Δ*φ*_*n*_| ~ SL	0.01	−0.08	−0.09	1.42	0.281	–	–	–

Correlations were estimated in each trial and then averaged. Estimates were made separately for the inner- (IN) and outer-sides (OUT) of the walker with respect to the direction of the turn. Hyphens replace large p-values.

**Table 3:** Correlation matrix of the gait parameters at the level of trial (n = 42) across seven participants and two repetitions in each of the three conditions.

	*M*	*SD*	ISI_IN_	CV_IN_	v_IN_	SL_IN_	*α*_OUT_	ISI_OUT_	CV_OUT_	v_OUT_	SL_OUT_
*α*_IN_	0.72	0.19	−0.09	−**0.40**^**†**^	**0.44**^**†**^	**0.52**^**‡**^	**0.95**^**‡**^	−0.09	**−0.43**^**†**^	**0.43**^**†**^	**0.50**^**‡**^
ISI_IN_	1.13	0.06		**0.55**^**‡**^	**−0.67**^**‡**^	0.01	−0.19	**1.00**^**‡**^	**0.58**^**‡**^	**−0.68**^**‡**^	0.02
CV_IN_	2.00	0.70			**−0.72**^**‡**^	**−0.48**^**†**^	**−0.41**^**†**^	**0.55**^**‡**^	**0.96**^**‡**^	**−0.72**^**‡**^	**−0.47**^**†**^
*v*_IN_	1.37	0.11				**0.73**^**‡**^	**0.46**^**†**^	**−0.68**^**‡**^	**−0.69**^**‡**^	**1.00**^**‡**^	**0.71**^**‡**^
SL_IN_	1.54	0.09					**0.45**^**†**^	0.01	**−0.41**^**†**^	**0.73**^**‡**^	**0.99**^**‡**^
*α*_OUT_	0.69	0.21						−0.19	**−0.51**^**‡**^	**0.43**^**†**^	**0.41**^**†**^
ISI_OUT_	1.13	0.06							**0.58**^**‡**^	**−0.68**^**‡**^	0.02
CV_OUT_	2.09	0.79								**−0.68**^**‡**^	**−0.39**^**†**^
*v*_OUT_	1.37	0.11									**0.72**^**‡**^
SL_OUT_	1.54	0.09									

*p < 0.05, ^†^*p* < 0.01, ^‡^*p* < 0.001.

**Table 4:** Parameters of the simple and mediated multilevel models for α with condition as predictor (exogenous variable) and centered velocity as mediator.

Effect	*α*_IN_				*α*_OUT_			
	Non-mediated		Mediated		Non-mediated		Mediated	
	Estimate	*SE*	Estimate	*SE*	Estimate	*SE*	Estimate	*SE*
Fixed effects								
*γ*_*α*_	**0.848**	**0.054**	**0.826**	**0.052**	**0.858**	**0.049**	**0.835**	**0.047**
*c*	−**0.131**	**0.030**			−**0.165**	**0.031**		
*γ*_*V*_			−0.005	0.019			−0.019	0.021
*a*			−**0.090**	**0.014**			−**0.090**	**0.016**
*b*			−**1.104**	**.456**			−**1.272**	**0.520**
*c*′ (direct effect)			−**0.232**	**0.052**			−**0.281**	**0.060**
*ab* (indirect effect)			**0.099**				**0.114**	
Model fit statistics								
AIC	−29.307		−128.900		−34.486		−113.900	
BIC	−18.881		−75.400		−24.060		−60.400	
Log-likelihood	20.653		86.500		23.243		79.000	
Deviance	−41.307		−172.900		−46.486		−157.900	
Groups (PP)	7.000		7.000		7.000		7.000	

Bold for significant fixed effect coefficients.
