# Supplementary material for: The role of environmental constraints in walking: Effects of steering and sharp turns on gait dynamics
Source: Sci Rep. 2016 Jun 27;6:28374. doi: 10.1038/srep28374 (PMC4937443; doi:10.1038/srep28374)
Supplement: Supplementary Information [file srep28374-s1.pdf]

The role of environmental constraints in walking: Effects of steering and sharp turns on gait dynamics

Dobromir G. Dotov*[1,2], Benoît G. Bardy[1,3], & Simone Dalla Bella[1,3,4,5]

[1]EuroMov, Université de Montpellier, Montpellier, France

[2]Centro de Ciencias de la Complejidad (C3), Universidad Nacional Autónoma de México, Ciudad de México

[3]Institut universitaire de France (IUF), Paris, France

[4]International Laboratory for Brain, Music, and Sound Research (BRAMS), Montreal, Canada

[5]Dept. of Cognitive Psychology, WSFiZ, Warsaw, Poland

* dobri.dotov@gmail.com

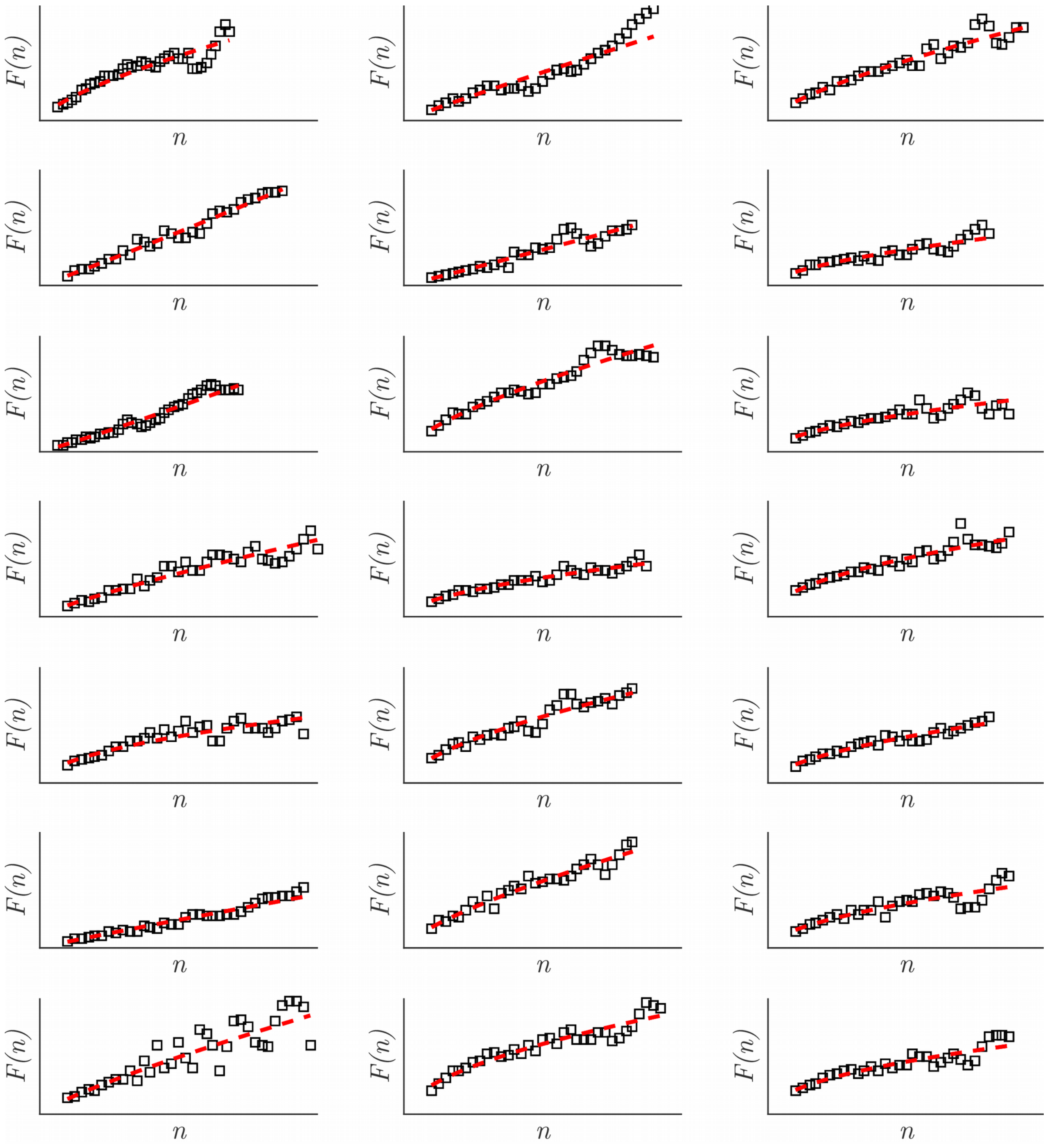

Figure S1. Individual trial fluctuation functions from DFA and corresponding fit for left turn trials. Rows correspond to participant and column to condition: unconstrained (left), constrained (middle), perturbed (right).

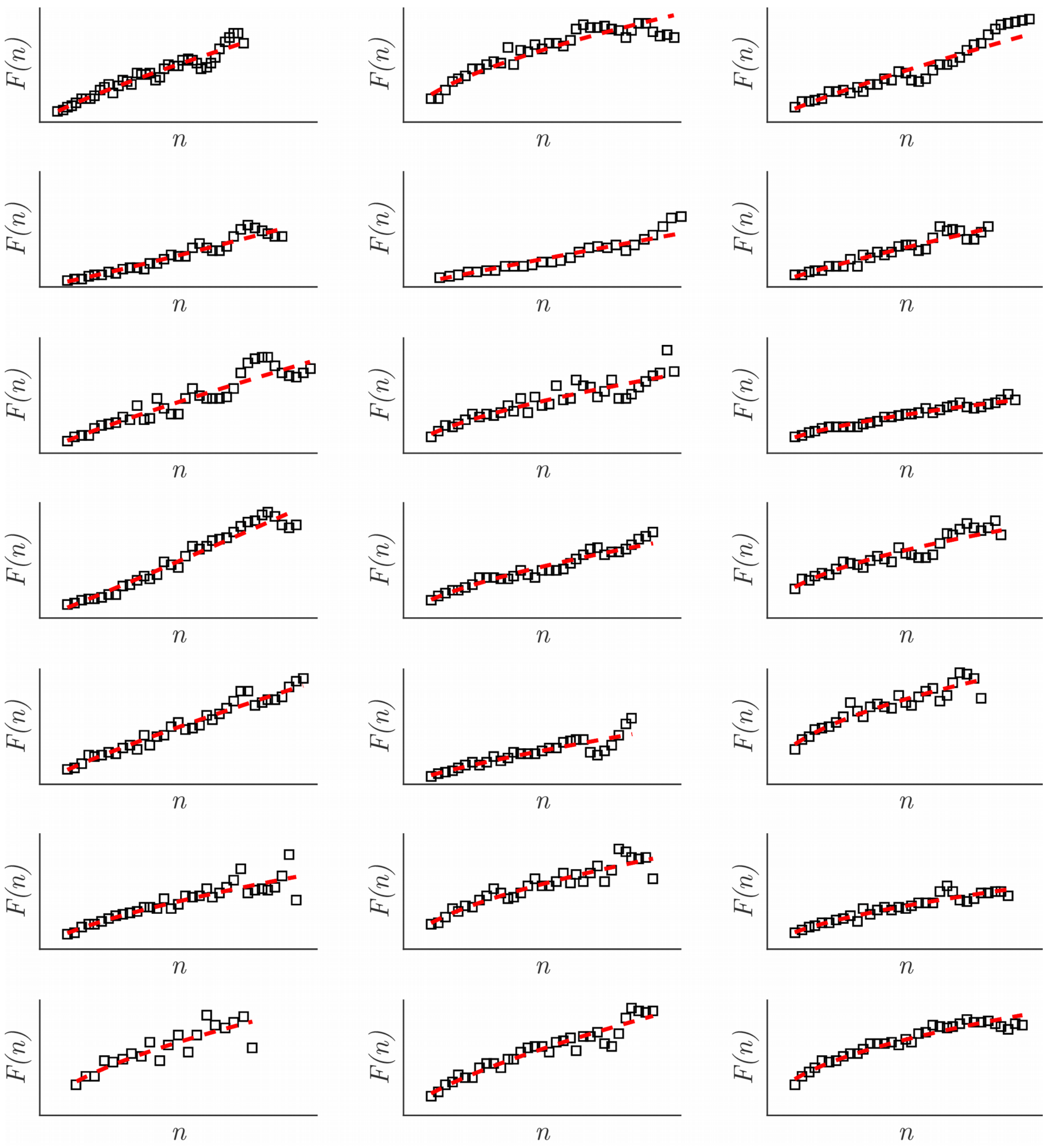

Figure S2. Individual trial fluctuation functions from DFA and corresponding fit for left turn trials. Rows correspond to participant and column to condition: unconstrained (left), constrained (middle), perturbed (right).
